# Improving the remote estimation of soil organic carbon in complex ecosystems with Sentinel-2 and GIS using Gaussian processes regression

**DOI:** 10.1007/s11104-022-05506-1

**Published:** 2022-06-03

**Authors:** Johanna Elizabeth Ayala Izurieta, Carlos Arturo Jara Santillán, Carmen Omaira Márquez, Víctor Julio García, Juan Pablo Rivera-Caicedo, Shari Van Wittenberghe, Jesús Delegido, Jochem Verrelst

**Affiliations:** 1 Image Processing Laboratory (IPL), University of Valencia, 46980 Paterna, Valencia Spain; 2 Faculty of Sciences, Escuela Superior Politécnica de Chimborazo, Riobamba, 060155 Ecuador; 3 Faculty of Natural Resources, Escuela Superior Politécnica de Chimborazo, Riobamba, 060155 Ecuador; 4 Faculty of Engineering, Universidad Nacional de Chimborazo, Riobamba, 060150 Ecuador; 5 Faculty of Forestry and Environmental Sciences, Universidad de Los Andes, Mérida, 5101 Venezuela; 6 Faculty of Science, Universidad de Los Andes, Mérida, 5101 Venezuela; 7 CONACYT-UAN, 63000 Tepic, Mexico

**Keywords:** Sentinel-2, Carbon stock mapping, Soil organic carbon (SOC), Gaussian processes regression, Vegetation indices, Ecuadorian páramo

## Abstract

**Background and aims:**

The quantitative retrieval of soil organic carbon (SOC) storage, particularly for soils with a large potential for carbon sequestration, is of global interest due to its link with the carbon cycle and the mitigation of climate change. However, complex ecosystems with good soil qualities for SOC storage are poorly studied.

**Methods:**

The interrelation between SOC and various vegetation remote sensing drivers is understood to demonstrate the link between the carbon stored in the vegetation layer and SOC of the top soil layers. Based on the mapping of SOC in two horizons (0–30 cm and 30–60 cm) we predict SOC with high accuracy in the complex and mountainous heterogeneous páramo system in Ecuador. A large SOC database (in weight % and in Mg/ha) of 493 and 494 SOC sampling data points from 0–30 cm and 30–60 cm soil profiles, respectively, were used to calibrate GPR models using Sentinel-2 and GIS predictors (i.e., Temperature, Elevation, Soil Taxonomy, Geological Unit, Slope Length and Steepness (LS Factor), Orientation and Precipitation).

**Results:**

In the 0–30 cm soil profile, the models achieved a R^2^ of 0.85 (SOC%) and a R^2^ of 0.79 (SOC Mg/ha). In the 30–60 cm soil profile, models achieved a R^2^ of 0.86 (SOC%), and a R^2^ of 0.79 (SOC Mg/ha).

**Conclusions:**

The used Sentinel-2 variables (FVC, CWC, LCC/C_ab_, band 5 (705 nm) and SeLI index) were able to improve the estimation accuracy between 3–21% compared to previous results of the same study area. CWC emerged as the most relevant biophysical variable for SOC prediction.

**Supplementary Information:**

The online version contains supplementary material available at 10.1007/s11104-022-05506-1.

## Introduction

Soil organic carbon (SOC) levels are directly related to the amount of soil organic matter (SOM), due to its composition of organic compounds that are highly enriched in carbon. SOM is a heterogeneous mixture of materials that range in stage of decomposition from fresh plant residues to highly decomposed material known as humus (Ontl and Schulte 2012). SOM contains roughly 55–60% C by mass. In many soils, C stored in SOM comprises most or all of the C stock – referred to as SOC – except where inorganic forms of soil C occur (FAO and ITPS 2015). A majority of studies since the last 120 years it is found that carbon comprises 58% of organic matter obtaining a conversion factor of 1.724 (Van Bemmelen 1890). However, the assumption that organic matter is 50% carbon, would in almost all cases, be more accurate (Pribyl 2010). The SOC pool is also a key indicator of soil quality as it affects essential biological, chemical and physical soil functions such as nutrient cycling, pesticide and water retention, and soil structure maintenance (Jandl et al. 2014). Moreover, the quantitative retrieval of SOC storage is of global interest due to its link with the carbon cycle, and the monitoring of soils with a large potential for carbon sequestration in the mitigation of climate change (Crowther et al. 2016; Zahasky and Krevor 2020; Tan et al. 2020). A pressing need is to reduce the human-caused elevated CO2 concentrations and to protect valuable natural carbon sinks such as preserving ecosystems with high SOC storage capacity (Meinshausen et al. 2009; Corbeels et al. 2019). On the other hand, ecosystems with spatial heterogeneity involve variations between land and atmospheric conditions (Adhikari et al. 2020), and SOC storage strongly relates to the physical, chemical and biological behavior of the soil (Mirchooli et al. 2020). The depletion of SOC stock is attributed to numerous factors including: decrease in the amount of biomass returned to the soil, change in soil moisture and temperature regimes (which accentuate the rate of decomposition of organic matter), high decomposability of crop residues due to differences in C:N ratio and lignin content, tillage-induced perturbations, decrease in soil aggregation and reduction in physical protection of the soil organic matter, and increase in soil erosion (Lal 2005, 2021). Therefore, SOC variations also are related to land use changes and events related to climate change (Grace et al. 2006; Martin et al. 2010; Müller-Nedebock et al. 2016; Zhou et al. 2019). Climatic changes such as the overall increasing temperatures tend to increase decomposition but this will be limited where soils become very dry (Keesstra et al. 2016), while in contrast, climate events such as extreme rainfall or higher temperature events may alter the micro-organisms/ biota and vegetation cover, leading to adverse effect over the mineralization processes (Lefévre et al. 2017). Hence, the magnitude of the effect of climate change over SOC stocks depends on the intricate interplay of local external factors, such as climate, and the ecosystem-specific composition of the organic matter itself that steers its interactions with the inorganic soil phase (Schmidt et al. 2011).

In the last years, some remote sensing techniques attempted to achieve SOC predictions with a high precision level (Angelopoulou et al. 2019) using high-spatial resolution spaceborne platforms such as Sentinel-2 (S2) (Vaudour et al. 2019). Through the European Union’s Copernicus programme free information services drawn from satellite Earth Observation and in-situ data can be obtained. Satellites from the Sentinel missions belong to a set of dedicated satellites developed and operated by the European Space Agency (ESA) and the European Organization for the Exploitation of Meteorological Satellites (EUMETSAT) (European Union 2015). Through its products, ESA aims to generate fully-calibrated estimates of at-land products quasi-independent of the original Earth Observation to offer aid and understanding of global carbon cycling (Plummer et al. 2006). The S2-A and S2-B satellites offer free multi-spectral decametric high-spatial resolution (10 to 60 m) optical imagery. From the S2 multi-spectral instrument (MSI) images biophysical products are routinely derived such as Fraction of Absorbed Photosynthetically Active Radiation (FAPAR), Leaf Area Index (LAI), Fractional Vegetation Cover (FVC), Leaf Chlorophyll Content (C_ab_) and Canopy Water Content (CWC), providing unique opportunities to exploit land products for complex ecosystems (ESA 2015; Hu et al. 2020). Due to the short revisit time and the spatial resolution of S2 it is possible to obtain a large availability of remote sensing data. Therefore, finding key S2 indicators that allows linking vegetation cover characteristics with SOC storage would open opportunities to improve the SOC variability description both at field and regional scales(Castaldi et al. 2019).

The estimation of SOC using remote sensing data would be a non-destructive and a non-invasive methodology. Important information can be obtained through the link between different environmental parameters and spectral information in the visible (400–700 nm) and near-infrared (700–2500 nm) regions (Sørensen and Dalsgaard 2005; Xie et al. 2011), where soil appears darker with increasing SOC content (Ladoni et al. 2010), and an increase of reflectance near 800 nm is identified with increasing SOM (Krishnan et al. 1980). Spectral absorption regions where C-rich plant constituents absorb can be related to the above soil characteristics (e.g. vegetation coverage and types of land uses) using various regression methods through a training Machine Learning Regression Algorithms (MLRA) (Verrelst et al. 2012).

Among the outstanding MLRA methods are: decision trees (Breiman 1996, 2001; Friedman 2001); geostatistics (Rasmussen 1996); Neural Networks (NN) (Haykin 1999); kernel-based methods (Suykens and Vandewalle 1999) and Gaussian Processes Regression (GPR) (Rasmussen and Williams 2006). GPR is a non-linear regression method that uses non-parametric Bayesian modelling (Rasmussen 1996; Rasmussen and Williams 2006) that is able to perform adaptive, nonlinear data fitting for complex datasets, using relevant bands during the development of a regression model. Therefore it is possible to explain the physical meaning of the model (Camps-Valls et al. 2009; Verrelst et al. 2012; Van Wittenberghe et al. 2014). GPR models can achieve high precision, as well as obtain confidence intervals in the predictions. Likewise, GPR methods have a great adaptability to data with low susceptibility to the presence of noise (Camps-Valls et al. 2009; Verrelst et al. 2013a). Hence, GPR methods applied to remote sensing of vegetation properties provide favorable results for vegetation traits such as LAI (Estévez et al. 2020; Verrelst et al. 2020; Zhang et al. 2020b). Also for the retrieval of soil properties, GPR methods obtained high accuracies to predict soil moisture content and soil water retention, while the retrieval of SOM is considered acceptable (Taneja et al. 2021; Rastgou et al. 2021). Models trained combining kriging and machine learning can also be used to reveal chemical soil properties related to the capacity of soils to store SOC (John et al. 2021). The quantification of global SOC storage and revealing of soils rich in SOC storage is a goal for mankind to generate preservations and monitoring strategies, carbon sequestration could potentially mitigate climate change (Crowther et al. 2016; Angelopoulou et al. 2019, 2020).

SOC is high at the upper soil layer and decreases with the depth (Jobbágy and Jackson 2000; Olson and Al-Kaisi 2015), but the topsoil layer is also more exposed to the effects of climatic changes, land uses changes, erosion, etc. Understanding the vertical distributions of SOC is key to predicting and simulating the influences of climate, global change, and human activities on the terrestrial carbon cycle (Wang et al. 2004). Differences in SOC accumulation also depend on the composition and age of the soil at different depths, therefore variables related with SOC storage would be more or less representative in the predicted models.

This study focuses on the largest herbaceous páramo ecosystem in Chimborazo province, Ecuador, (MAE 2013), and wherefore we aim to develop a regression model to predict SOC at different soil depths/layers with a high level of precision based on an in situ validated GPR method. The objectives are: (i) to assess several variables linked to SOC sequestration to calibrate a prediction model optimized with GPR for estimation of SOC at different soil depths (0–30 cm and 30–60 cm below ground), and (ii) to spatially predict and compare the SOC content at high spatial resolution at 0–30 cm and 30–60 cm soil profiles.

## Materials and methods

### Study area

The páramo ecosystem forms a North–South corridor of more than 2000 km between Venezuela and Peru, in one of the most dynamic mountain ranges, geologically and biogeographically speaking (Jorgense and Ulloa 1994; Hofstede 2004). With an elevation generally between 3000 and 4000 m a.s.l and topographic heterogeneity, it is the largest supplier of water to the Andes of Venezuela, Colombia and Ecuador and to extensive parts of the inter-Andean areas, from the Caribbean and Pacific coasts of Costa Rica and Panama, to the desert of northern Peru (Hofstede et al. 2014). In Ecuador, the páramo covers the upper part of the two mountain ranges that run north–south, where the soils principally correspond to the volcanic origin of different ages. The main difference between the soils of the páramo is due to the difference in bedrock between the north and the south of the country. The ecosystem is characterized by its low temperatures, a high humidity despite moderate rainfall and weak evaporation, and a high capacity of water retention and regulation. It has high concentrations of SOM and their soils are deep (up to several meters) with variations influenced by soil evolution (Mena et al. 2000). Consequently, the total amount of carbon stored per hectare of páramo can be greater than that of a tropical forest soil, hence the páramo soils are considered as SOC sink (Hofstede 2004). Studying this type of ecosystem and revealing the SOC reserves with a high level of precision could contribute to the understanding of global SOC distribution.

**Fig. 1:**
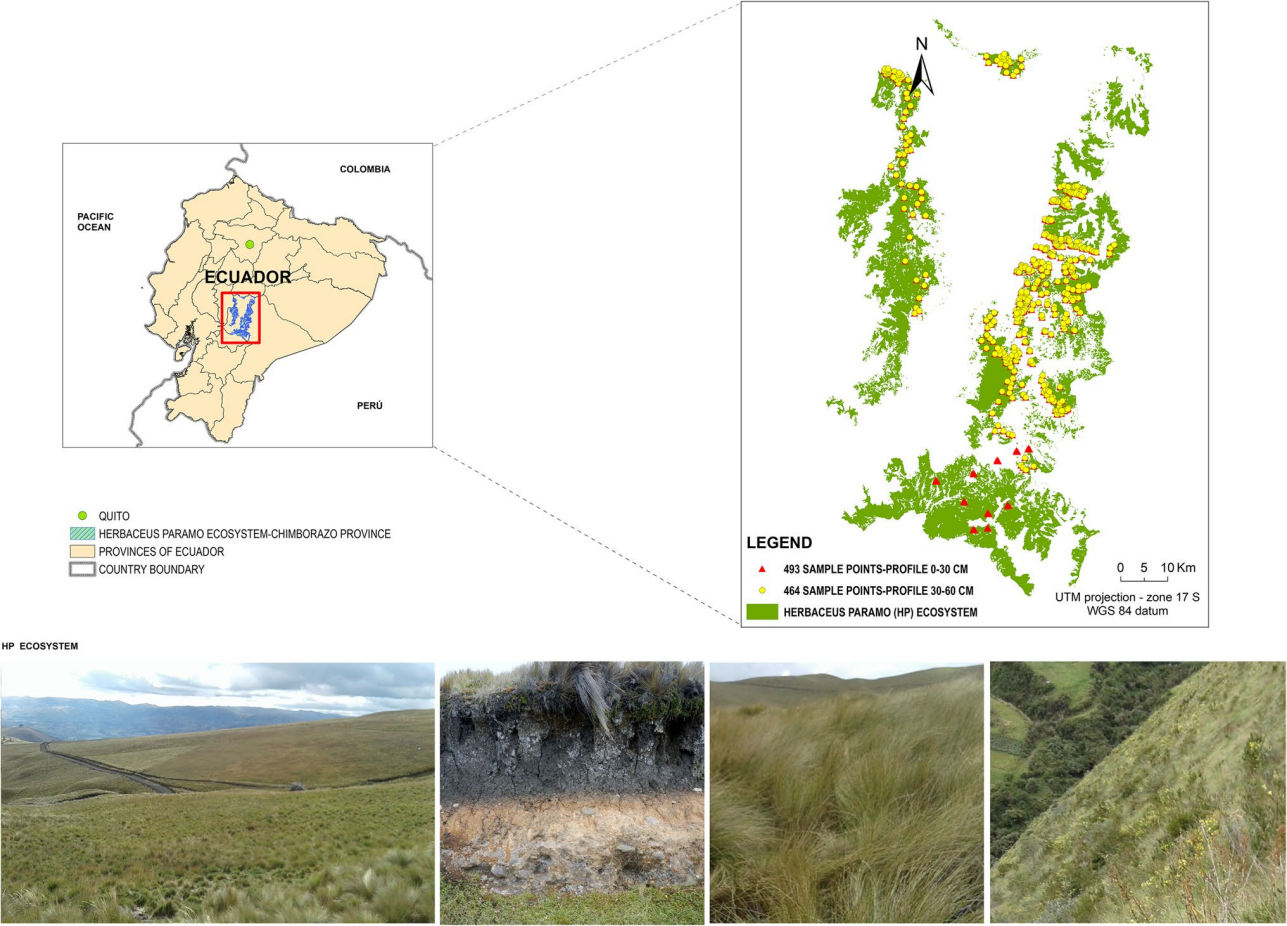
Study area and distribution of the SOC sampling points

The study area is the herbaceous páramo ecosystem in the central Ecuadorian páramo region. It is a complex mountainous system with an elevation range between 2303–4501 m a.s.l. and covering 25.7% of the Chimborazo province (Fig. 1). Extended between 78º39' west longitude and 1º39' south latitude, the study area covers approximately 1667.6 km^2^ (Ayala Izurieta et al. 2021). The ecosystem boundary is the Western and Eastern Cordilleras arranged in meridian direction (Moreno et al. 2018).

The weather is cold-humid with a constant humidity, and a mean annual temperature of 11 ºC. The cloud cover in the study area is high most of the time due to the high level of humidity and the mountainous conditions. About the climate, it is quite stable along the year, with larger temperature changes during the day compared to the annual changes of the mean temperature. Therefore dry and wet seasons occur but without notable differences (Mena Vásconez et al. 2011).

The herbaceous páramo soils are characterized by a soil water retention and regulation capacity between 0.55 and 0.90 cm^3^ cm^−3^ (Ministerio de Ambiente del Ecuador 2012; Guio Blanco et al. 2018; Moreno et al. 2018). Additionally, these soils are resistant to erosion with a stable structure, micro-aggregation, high porosity, high humidity rate and permeability, stimulating the root development (Mena et al. 2000). The predominant vegetation is mainly based on tufted grasses larger than 50 cm in height. Within the dominant species, the genus *Calamagrostis*, *Agrostis*, *Festuca*, *Cortaderia* and *Stipa* stand out, shrub patches of the genus *Diplostephium*, *Hypericum* and *Pentacalia* are also differentiated, among others (MAE 2013).

### Soil organic carbon data

A database with SOC data on two soil profiles (0–30 cm and 30–60 cm) collected in 2016 and 2017 was used. The initial database contains around 500 in situ SOC sampling points, with a random spatial sampling distribution taking into account the geological units, the taxonomy of the soil, and accessibility based on the steepness of the terrain and availability of ballast roads and trails (Ayala Izurieta et al. 2021).

The in situ soil samples were collected from the top layer (0 to 30 cm) and 30 to 60 cm below ground using a blast-hole, then these are stored and taken to the laboratory for SOC quantification processes. Soil samples were sieved (2-mm mesh), oven-dried at 105 °C, and ground prior to analysis. The total SOC in the collected soil samples were determined with an Elemental Analyzer (Flash 2000 Organic Elemental Analyzer type CHNS/O, ThermoFicher Scientific). Specifically, a soil aliquot, containing approximately 10 mg of organic carbon in silver capsules, was weighed (Tonon et al. 2010; Bateni et al. 2021). Additional samples were taken at each location of the sampling points of both profiles to determine the soil bulk density (in g/cm^3^). Cylinders of 88 cm^3^ were used, taking undisturbed soil samples (Al-Shammary et al. 2018) and then the common volume cylinder method was applied (Bongiorno et al. 2019). With the soil sample SOC weight % obtained (g C/100 g of soil) and the soil bulk density of the sample, the SOC content was expressed in Mg/ha (Lee et al. 2009; Tonon et al. 2010; Al-Shammary et al. 2018; Bongiorno et al. 2019; Ayala Izurieta et al. 2021; Bateni et al. 2021).

After identifying the anomalous data points (points extremely far from the tendency line and points without information or zero value), 493 and 464 SOC data points were kept in the 0–30 cm and 30–60 cm soil profile datasets, respectively (see Fig. 1). The geographical position (UTM coordinates, datum WGS84) of each data point was recorded (using a PGS-Trimble JUNO SB handheld with 2-to- 5-m positional accuracy in real time) to georeference the SOC data and to obtain a spatial relation with all variables evaluated on this study to be potential proxies of SOC content.

### Meteorological and geophysical data

Considering the environmental factors, the following variables were analysed: temperature, elevation, soil taxonomy, geological unit, slope length and steepness (LS Factor), orientation and precipitation. In a previous study these variables were identified as being related to the storage of SOC in different levels of importance (Ayala Izurieta et al. 2021). The air temperature and precipitation are linked with SOC decomposition rates (Zhang et al. 2015). Also, soils with lower decomposition rates of carbon have high field water holding capacity (WHC) (Xu et al. 2016). The average annual temperature and the precipitation data in the herbaceous páramo ecosystem in 2015 were obtained with a kriging interpolation method (Oliver and Webster 2007; Mishra et al. 2009) using the data from 57 meteorological weather stations (21 stations belonging to Institute of Meteorology of Ecuador (INAMHI) located within Chimborazo province, 3 stations belonging to National University of Chimborazo (UNACH), to cover the southeast of Chimborazo province and 33 stations belonging to INAMHI located in surrounding areas out of the study area).

Topographic factors can be indicators of SOC due to their influence on the capacity of the soil to store SOC (Abebe et al. 2020). The uneven soil topography is typical of this complex geographic area (Podwojewski and Poulenard 2004), therefore elevation and orientation variables are evaluated. Also, due to the strong elevation gradients, land-use changes and soil erosion is considered an important factor for the SOC variability, as changes in the soil erosion can alter long-term stored SOC in the top soil layers and lead loss of SOC (Olson et al. 2016; Shi et al. 2020). The LS factor (Desmet and Govers 1996; Panagos et al. 2015; Lu et al. 2020) is one of the most important controlling factors of soil characteristics and geomorphic processes such as soil erosion (Khanifar and Khademalrasoul 2020). It is the product between the L and S factors (Eqs. 1 to 6, see below), where factor L correspond to the influence of impact of the slope length while the factor S explains the effect of the slope's inclination (Ozsoy et al. 2012; da Cunha et al. 2017). The LS factor map was created in ArcGis 10.2 software using the DEM corresponding to the study area with a spatial resolution of 30 m and applying the expressions (1) to (6), where A_i,j_ is the accumulation area with coordinates (i,j) [m^2^]; D is the length of the pixel size [m]; x is the shape coefficient [dimensionless]; m has values between 0 and 1 [dimensionless]; $\theta$ is the slope angle [rad]; and $\beta$ is the ratio of rill to interrill erosion [dimensionless] (Desmet and Govers 1996).

1 $$LS=L*S$$

2 $$\beta =\frac{\frac{\mathrm{sin}\theta }{0.0896}}{{3\mathrm{sin}\theta }^{0.8}+0.56}$$

3 $$m=\frac{\beta }{\beta +1}$$

4 $$L=\frac{{\left[{A}_{i,j}+D\right]}^{(m+1)}-{{A}_{i,j}}^{(m+1)}}{{x}^{m}{D}^{m+2}{(22.13)}^{m}}$$

5 $$\;\begin{array}{ccc}S=10.8\;\sin\;\theta+0.03,&\mathrm{if}&tg\;\theta<0.09\end{array}$$

6 $$\begin{array}{ccc}S=16.8\;\sin\;\theta-0.05,&\mathrm{if}&tg\;\theta\geq0.09\end{array}$$

Due to topographic heterogeneity of this mountain geosystem and taking into account that the soil use of herbaceous páramo has only natural vegetation, the soil classification is also evaluated as a predictor based on the Soil Taxonomy and Geological Unit. These variables are highly relevant for the predictive modelling of SOC storage (Ayala Izurieta et al. 2021). The national geological mapping classification was used, where 31 geological units conform to the herbaceous páramo ecosystem (SNI 2011). On the other hand, the Soil Taxonomy is a more international system of classification, wherefore the USDA Soil Taxonomy is used (USDA and NRCS 2014).

### Image processing and remote sensing data

Given its good spatial resolution, we selected multispectral S2 imagery for this study. Ten S2 bands were used, i.e., four bands at 10 m (B2-490 nm, B3-560 nm, B4-665 nm, B8-842 nm) and six bands at 20 m (B5-705 nm, B6-740 nm, B7-783 nm, B8a-865 nm, B11-1610 nm, B12-2190 nm) (ESA 2015). There are three bands in the visible spectrum, a band to the near infrared, four bands located in the red edge and two bands of SWIR. S2 has a temporal resolution of 5 days with both satellites operating (S2-A, S2-B), or 10 days referring to a single satellite. The S2 images used are Level-1C processing products (ESA 2015) (see Table 1), downloaded from Global Visualization Viewer (GloVis) web service of the United States Geological Survey (USGS 2017).

**Table 1:** Sentinel-2 images used

S2 image ID	Tile number	Tile extend	Date	% CC^a^	Platform	Projection
L1C_T17MQU_A010218_20170606T154217	T17MQU	100 km	06/06/2017	23.88	S2-A	UTM Zone 17S Datum GS84
L1C_T17MQT_A010218_20170606T154217	T17MQT		06/06/2017	25.72		
L1C_T17MQU_A014651_20180412T153620	T17MQU		12/04/2018	11.39		
L1C_T17MQT_A014651_20180412T153620	T17MQT		12/04/2018	13.77		

^a^ Cloud cover (CC) percentage

**Fig. 2:**
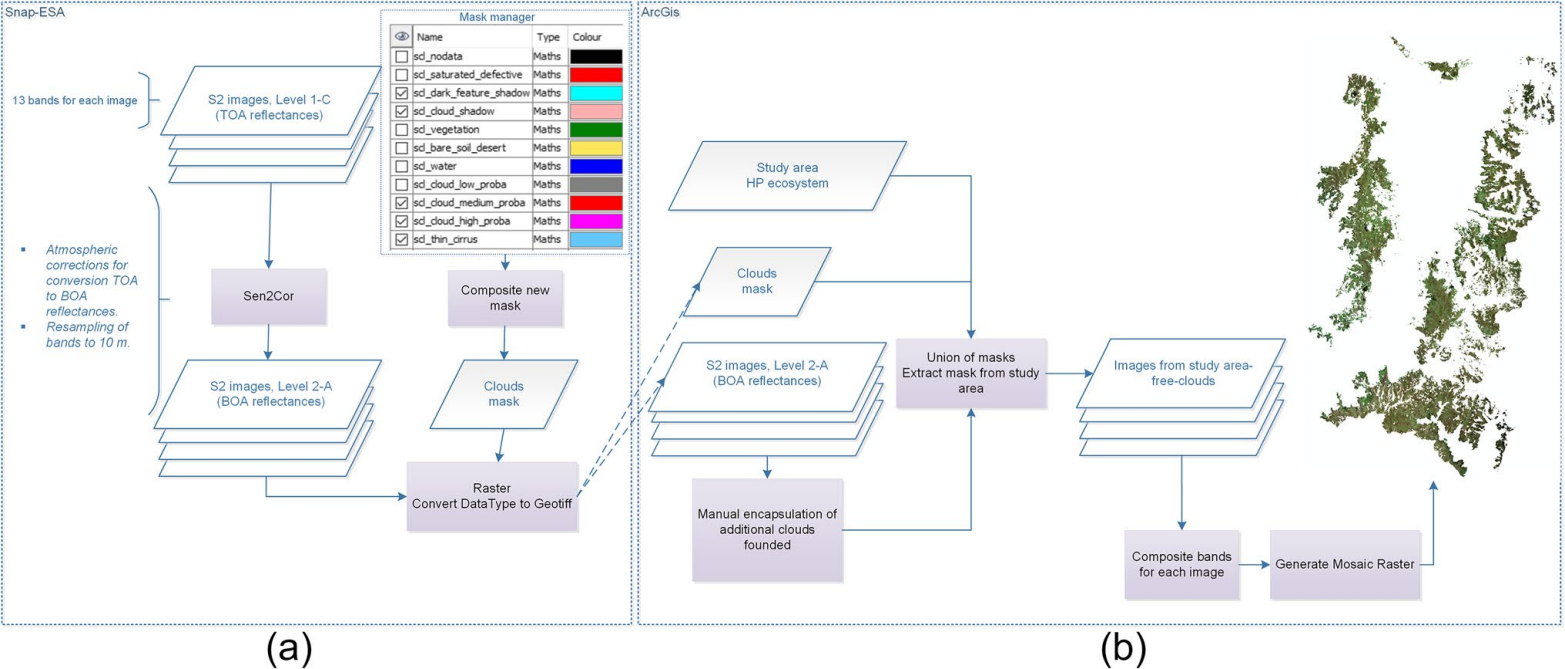
**a** Atmospheric correction and creation of clouds mask based on additional products from S2, **b** Process to eliminate clouds and creation of final mosaic

Level-1C products include radiometric and geometric corrections, and also orthorectification and spatial registration in a global reference system using a Digital Elevation Model (DEM) to project the image in cartographic coordinates (ESA 2015), leading to Top Of Atmosphere (TOA) reflectances. To obtain per-pixel Bottom Of Atmosphere (BOA) reflectances, i.e. processing Level 2A, the Sen2Cor Toolbox version 2.4 through the Sentinel Applications Platform (SNAP) software version 5.0 was used (see Fig. 2a). The complex study area is typically characterized by a high percent of cloud cover, which demands for a special treatment for encapsulation and removal of clouds. The clouds encapsulation was realized using the additional Cloud Mask products combined with manual identifying and removing of clouds. Finally, a BOA mosaic of the herbaceous páramo ecosystem was obtained with ArcGis 10.2 software (see Fig. 2b).

This study uses the S2 spectral indices NDVI, SAVI, WDRVI, EVI2, NDWI, VARIg, NDSI, BI, NDMI, NBR, NBR2 and adds a physiologically-based green leaf area index (LAIgreen index) known as the Sentinel-2 LAIgreen Index (SeLI) (Pasqualotto et al. 2019) (see Table 2). The SeLI index has the potential to be used in a unified algorithm for LAIgreen estimation or to identify bare areas, wherefore it can provide relevant information as a SOC storage indicator.

**Table 2:** S2-Spectral indices evaluated in the regression model

Index/Reference	Formula	Formula with specific S2 bands
Sentinel-2 LAIgreen Index (SeLI) (Pasqualotto et al. 2019)	$\mathrm{SeLI}=\frac{\mathrm{NIR}-\mathrm{R}}{\mathrm{NIR}+\mathrm{R}}$	$\mathrm{SeLI}=\frac{\mathrm{B}8\mathrm{A}-\mathrm{B}5}{\mathrm{B}8\mathrm{A}+\mathrm{B}5}$
Normalized Difference Vegetation Index—NDVI (Rouse et al. 1974)	$\mathrm{NDVI}=\frac{\mathrm{NIR}-\mathrm{R}}{\mathrm{NIR}+\mathrm{R}}$	$\mathrm{NDVI}=\frac{\mathrm{B}8-\mathrm{B}4}{\mathrm{B}8+\mathrm{B}4}$
Soil-Adjusted Vegetation Index—SAVI (Huete 1988), L value according (Ayala et al. 2017)	$\mathrm{SAVI}=\frac{\mathrm{NIR}-\mathrm{R}}{\mathrm{NIR}+\mathrm{R}+\mathrm{L}}\left(1+\mathrm{L}\right)$ $\mathrm{L}=0.15$	$\mathrm{SAVI}=\frac{\mathrm{B}8-\mathrm{B}4}{\mathrm{B}8+\mathrm{B}4+0.15}\left(1+0.15\right)$
Wide Dynamic Range Vegetation Index – WDRVI (Gitelson 2004), a value according (Ayala et al. 2017)	$\mathrm{WDRVI}=\frac{\mathrm{aNIR}-\mathrm{R}}{\mathrm{aNIR}+\mathrm{R}}$ $\mathrm{a}=0.05$	$\mathrm{WDRVI}=\frac{0.05\mathrm{ B}8-\mathrm{B}4 }{0.05\mathrm{ B}8+\mathrm{B}4}$
Enhanced Vegetation Index 2 – EVI2 (Jiang et al. 2008)	$\mathrm{EVI}2=2.5 \frac{\mathrm{NIR}-\mathrm{R}}{\mathrm{NIR}+2.4\mathrm{ R}+1}$	$\mathrm{EVI}2=2.5 \frac{\mathrm{B}8-\mathrm{B}4}{\mathrm{B}8+2.4\mathrm{ B}4+1}$
Normalized Difference Water Index—NDWI (McFeeters 1996)	$\mathrm{NDWI}=\frac{\mathrm{G}-\mathrm{NIR}}{\mathrm{G}+\mathrm{NIR}}$	$\mathrm{NDWI}=\frac{\mathrm{B}3-\mathrm{B}8}{\mathrm{B}3+\mathrm{B}8}$
Visible Atmospherically Resistant Vegetation Index green – VARIg (Cammarano et al. 2014; Gitelson et al. 2002)	${\mathrm{VARI}}_{\mathrm{G}}=\frac{\mathrm{G}-\mathrm{R}}{\mathrm{G}+\mathrm{R}}$	${\mathrm{VARI}}_{\mathrm{G}}=\frac{\mathrm{B}3-\mathrm{B}4}{\mathrm{B}3+\mathrm{B}4}$
Normalized Difference Snow Index – NDSI (Takeuchi and Yasuoka 2004)	$\mathrm{NDSI}=\frac{\mathrm{SWIR}1-\mathrm{NIR}}{\mathrm{SWIR}1+\mathrm{NIR}}$	$\mathrm{NDSI}=\frac{\mathrm{B}11-\mathrm{B}8}{\mathrm{B}11+\mathrm{B}8}$
Bare Soil Index- BI (Chen et al. 2004)	$\mathrm{BI}=\frac{\left(\mathrm{SWIR}1+\mathrm{R}\right)-(\mathrm{NIR}+\mathrm{B})}{\left(\mathrm{SWIR}1+\mathrm{R}\right)+(\mathrm{NIR}+\mathrm{B})}$	$\mathrm{BI}=\frac{\left(\mathrm{B}11+\mathrm{B}4\right)-(\mathrm{B}8+\mathrm{B}2)}{\left(\mathrm{B}11+\mathrm{B}4\right)+(\mathrm{B}8+\mathrm{B}2)}$
Normalized Difference Moisture Index – NDMI (Wilson and Sader 2002; Hislop et al. 2018)	$\mathrm{NDMI}=\frac{\mathrm{NIR}-\mathrm{SWIR}1}{\mathrm{NIR}+\mathrm{SWIR}1}$	$\mathrm{NDMI}=\frac{\mathrm{B}8-\mathrm{B}11}{\mathrm{B}8+\mathrm{B}11}$
Normalized Burn Ratio – NBR (Key and Benson 2006)	$\mathrm{NBR}=\frac{\mathrm{NIR}-\mathrm{SWIR}2}{\mathrm{NIR}+\mathrm{SWIR}2}$	$\mathrm{NBR}=\frac{\mathrm{B}8-\mathrm{B}12}{\mathrm{B}8+\mathrm{B}12}$
Normalized Burn Ratio 2- NBR2 (Stokey et al. 2016)	$\mathrm{NBR}2=\frac{\mathrm{SWIR}1-\mathrm{SWIR}2}{\mathrm{SWIR}1+\mathrm{SWIR}2}$	$\mathrm{NBR}2=\frac{\mathrm{B}11-\mathrm{B}12}{\mathrm{B}11+\mathrm{B}12}$

Punctual spectral information (i.e., individual S2-bands, spectral indices) is extracted from the final mosaic corresponding to the SOC sampling databases (493 SOC data points in the 0–30 cm profile and 464 SOC data points in the 30–60 cm profile); this database is used for training the regression model for SOC prediction. It contains spectral information of the input variables to be evaluated as predictors (S2-bands), spectral indices, meteorological and soil data, and also biophysical data). In the same way, the final mosaic is used to create an extensive geo-database of points, one point per pixel in the study area. The database contains information on the found SOC predictors, and it is used to obtain the SOC prediction values using the trained and optimized regression model.

### Biophysical vegetation data

S2-derived vegetation biophysical variables were directly obtained from an automatic process implemented in SNAP 5.0 including FAPAR, LAI, FVC, LCC/C_ab_ [µg.cm^−2^] and CWC [mg.cm^−2^] for each L2A product of the used scenes (Table 1). These biophysical parameters were evaluated as variables linked to the SOC storage. FAPAR is the fraction of incoming solar radiation in the range of 400–700 nm that is absorbed by vegetation. It characterizes the growth status of the vegetation and it can be used for tracing mass and energy exchanges (Chen et al. 2020). LAI parameter is the one-sided green leaf area per unit ground area (Chen and Black 1992), related to vegetation growth and productivity, as well as significant for modelling energy fluxes, water and carbon (Chen et al. 2002; Verrelst et al. 2015). FVC is the ratio of the vertically projected area of vegetation to the total surface area (Song et al. 2017) and is used to separate vegetation and soil in energy balance processes, including temperature and evapotranspiration. FCV is also used for monitoring of green vegetation, due to it does not depend on variables such as the geometry of illumination as compared to FAPAR (Weiss et al. 2000; Weiss and Baret 2016). LCC or C_ab_ is a pivotal parameter in the monitoring of agriculture and carbon cycle modelling at regional and global scales as it can provide crucial information for understanding photosynthesis potential (Osborne and Raven 1986) plant stress (Carter 1994) and physiological status (Qian and Liu 2020; Zhang et al. 2020a). CWC is the product of LAI with leaf canopy content (LWC) (Verrelst et al. 2020), LWC is the Leaf water content and it has important physiological and ecological significance for plant growth (Wang et al. 2021).

Finally, the cloud removal step was also applied to the vegetation product images (see Fig. 2b). Information of biophysical variables was added to each pixel of the database for SOC prediction, as well as to the sampling point database for training the SOC regression model.

### Machine learning regression method—Gaussian Process Regression

**Fig. 3:**
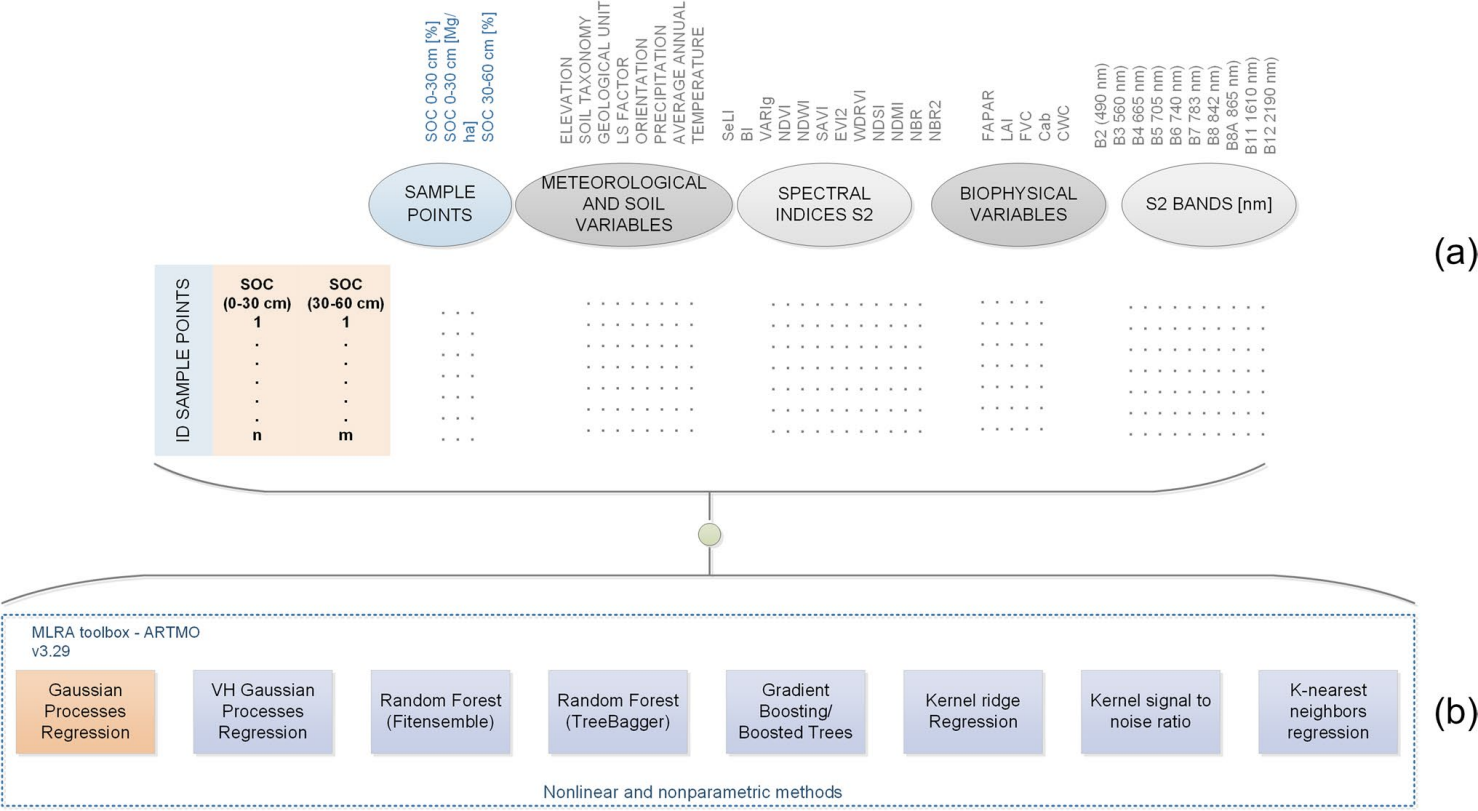
**a** Initial database for training, **b** Regression methods proved using the initial database for training

Unlike class labels obtained through a classification process, a regression produces predictions of continuous values. Considering that SOC database contains in situ sampling campaigns as well as meteorological information and the corresponding biophysical, spectral and soil surface characteristics of the study area, then a non-parametric regression model is applied. It allows to achieve a SOC prediction model adjusted with respect to the information of the input variables and existing SOC observations (see Fig. 3).

In order to probe the GPR capacity, multiple machine learning regression algorithms (MLRA) are used. For this purpose, several available methods were applied using the ARTMO software (Automated Radiative Transfer Model Operator) version 3.29 (Verrelst et al. 2019). Eight regression methods were tested (see Fig. 3b) using the same training data (input variables / bands, SOC data in situ) with a data percentage of 70% for training and 30% for validation.

### SOC prediction with Gaussian Processes Regression (GPR)

Previous studies with GPR for the evaluation of biophysical parameters obtained results superior to those obtained with other non-parametric regression methods such as NN and KRR (Verrelst et al. 2012). GPR combining remote sensing data may be suitable for predicting physicochemical parameters of the soil (Rasmussen and Williams 2006; Verrelst et al. 2013a) as is the case of the SOC in this study, obtaining predictions with a resolution level of pixel size.

A Gaussian Process (GP) defines a distribution over functions. In other words, it generates a finite set of random variables, of which there is a joint Gaussian distribution; GPR is a non-linear regression method that uses non-parametric Bayesian modelling that considers the variance of the data set and a maximization of the probability margin in the training set using a scaled anisotropic Gaussian kernel function (see Table 3) (Pérez-Planells et al. 2015). GPR allows identifying the important characteristics of the input variables (Rasmussen and Williams 2006) and evaluating the relative contribution highlighting the most relevant bands or parameters to the prediction model. In this way, it is possible to find the input variables linked to SOC sequestration (SOC predictors) in order of importance for their contribution to the optimization of the resulting model.

**Table 3:** Mathematical expressions of the GPR method (Rasmussen and Williams 2006; Camps-Valls et al. 2009; Van Wittenberghe et al. 2014)

Mathematic expression	Detail
The relationship between the input variable (B-variables) $x\in {\mathbb{R}}^{B}$ and the output variable (SOC) $y\in {\mathbb{R}}$: $\widehat{y}=f\left(x\right)=\sum_{i=1}^{N}{\propto }_{i}K({x}_{i},\mathrm{x})$	${(X)}_{i=1}^{N}$: input bands or variables used in training ${\propto }_{i}$: weights assigned to the training bands or variables $K$: sophisticated kernel function that assesses the similarity between the test variable and all N bands or training variables (Camps-Valls et al. 2009; Verrelst et al. 2012, 2013a)
Scaled anisotropic Gaussian kernel function: ${K}_{(Xi,Xj)}=v \mathrm{exp}(-{\sum }_{b=1}^{B}\frac{{({x}_{i}^{b}-{x}_{j}^{b})}^{2}}{{2\sigma }_{b}^{2}})$	ʋ: scale factor B: number of variables (bands) ${\upsigma }_{b}$: factor that controls the propagation of the relationship for each of the input variables *b* (Verrelst et al. 2013a, b; Van Wittenberghe et al. 2014)

Method parameters $v, {\upsigma }_{b}\mathrm{ and }{\propto }_{i}$ are automatically optimized using marginal likelihood on the training set (Rasmussen and Williams 2006; Camps-Valls et al. 2009; Van Wittenberghe et al. 2014). The ${\propto }_{i}$ values indicate the relevance of each input band x_i_. The mean SOC prediction comes from the weighted average of the values of the SOC parameters of interest related to the training samples closest to the analysis sample. The inverse of ${\upsigma }_{b}$ indicates the relevance of each $b$ band, i.e., low values of this parameter indicate that $b$ band provides more information to the training function $K$ (Verrelst et al. 2013b).

### GPR model training, optimization and calibration process

**Fig. 4:**
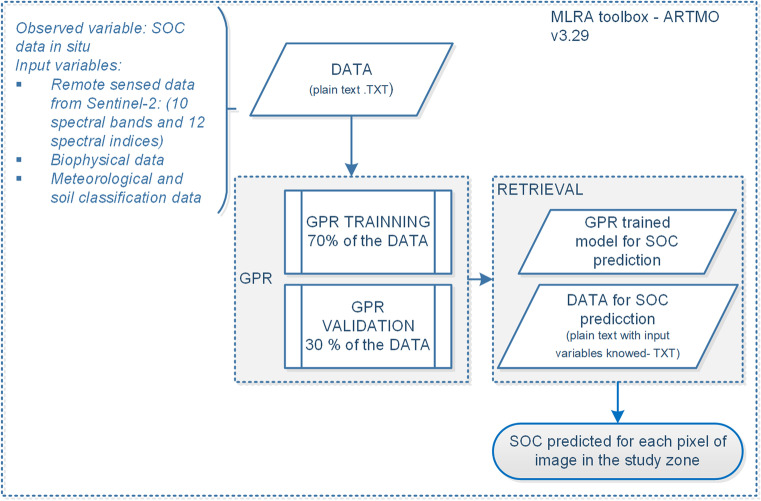
Processes diagram for GPR with ARTMO

After testing all input variables as SOC predictors (see Fig. 3) with eight MLRA models, the best model and input variables (*b*) are selected and used to calibrate the GPR final model. The procedural diagram followed for training of the SOC prediction model is observed in Fig. 4, the model is trained as well as used to predict the SOC content in the herbaceous páramo ecosystem using ARTMO 3.29 software. The SOC database used contains SOC information in two profiles, 493 SOC sampling points corresponding to profile 0–30 cm below ground, and 464 points from the profile 30–60 cm below ground, in % and Mg/ha units.

The training process uses 70% of in situ SOC samples database and 30% of data are reserved for model validation. When the R^2^ reaches a maximum value and also the RMSE is reduced, then input variables are defined as predictors. The ${\upsigma }_{b}$ value expresses the relationship for each of the input variables. A SOC predictor model is obtained for each profile or horizon (0–30 cm and 30–60 cm) and used for the prediction process (retrieval), as illustrated in Fig. 4. The spatial structure of S2-images is used to generate bands for all SOC predictors obtained in the GPR process with the same spatial resolution (10 m). Its file is used to predict SOC for each pixel in the 0–30 cm profile and 30–60 cm profile.

Spectral band relevance is obtained for each input band ${\upsigma }_{b}$ is observed to obtain a good model calibration. In order of comparing the importance variables for each profile (0–30 cm and 30–60 cm) in both units (SOC% and SOC Mg/ha), polar plots were obtained analyzing b. In the polar plots are possible obtain a positive representation of the spectral relevance with a conversion of lower values of ${\upsigma }_{b}$ into higher ones and calculating the relative ${\upsigma }_{b}$ (Peppo et al. 2021). In this study, the polar plots used also introduce a function of scaling in order to detect relevance differences between bands, this is detailed in expression (7) (Peppo et al. 2021) and for the graphical representation the expression (8) is used.

7 $${\upsigma }^{2}=\left(1-\left(\frac{{\upsigma }^{2}}{\mathrm{max}{\upsigma }^{2}}\right)/\left(sum{\upsigma }^{2}\right)\right)*100$$

8 $${\upsigma }^{2}={log}_{10} \left(\frac{{\upsigma }^{2}}{\mathrm{max}{\upsigma }^{2}}\right)+1$$

## Results

**Fig. 5:**
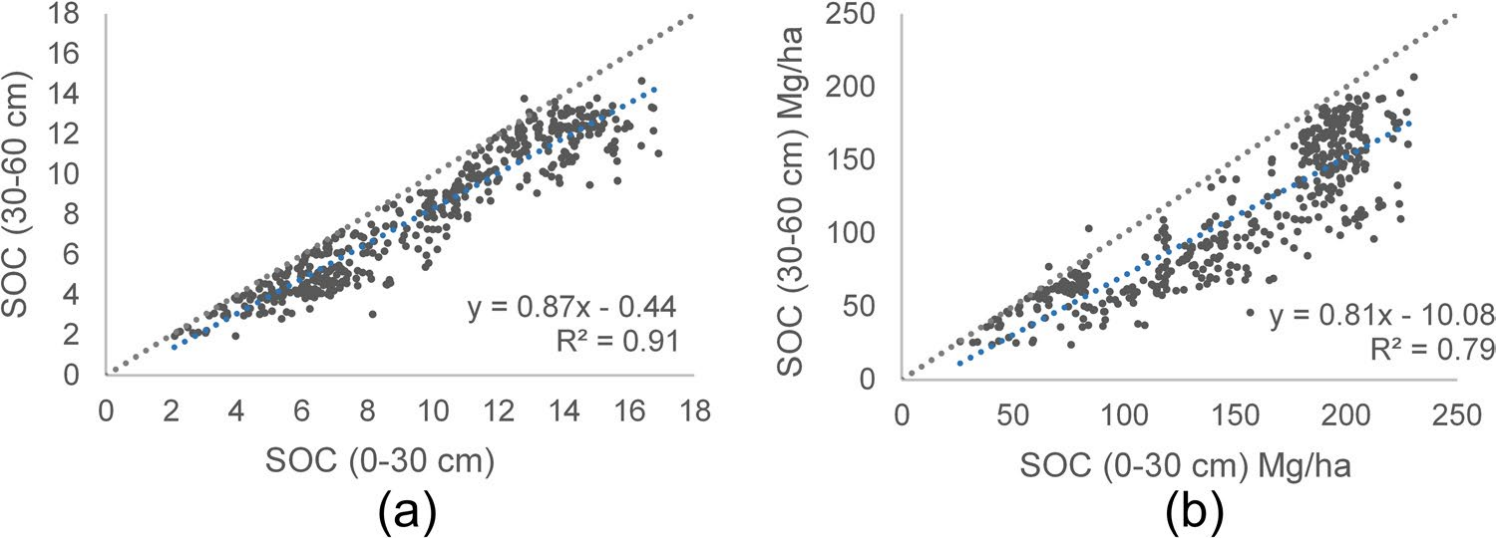
Scatterplots comparing profiles of SOC distribution of sample data points. **a** 0–30 cm profile vs 30–60 cm profile-%, **b** 0–30 cm profile vs 30–60 profile-Mg/ha. Line 1:1 and tendency line (in blue) are shown

Comparing the SOC sample data points of both depths, we note a decrease of SOC storage in the 30–60 cm profile (see Fig. 5). The SOC prediction models for 0–30 cm and 30–60 cm profiles were done, using the training data base with 493 SOC sampling data points from 0–30 cm profile and 494 SOC data points from 30–60 cm profile (in weight % and in Mg/ha). The importance level of meteorological variables, soil (topographic, geological-taxonomic) and S2 variables (10 bands, 12 indices and 5 biophysical variables (FAPAR, LAI, FVC, C_ab_, CWC)) for SOC prediction were founded using various regression methods in ARTMO-MLRA. With 70% of the data for training and 30% for validation, the goodness-of-fit statistics were shown in Table 4, observing that GPR has higher precision with a higher R^2^ between 3–7% with respect to the second most relevant method. Therefore, the GPR method was identified as the most suitable method for SOC prediction in the study zone.

**Table 4:** Comparative performance results of the regression methods analyzed, using all the variables to predict SOC. The best performing method is bolded

Method SOC % 0–30 cm	R^2^, RMSE	Method -SOC Mg/ha 0–30 cm	R^2^,RMSE	Method SOC % 30–60 cm	R^2^, RMSE	Method SOC Mg/ha 30–60 cm	R^2^, RMSE
***GPR***	**0.81, 1.79**	***GPR***	**0.76, 25.91**	***GPR***	**0.86, 1.24**	***GPR***	**0.80, 20.83**
VH GPR	**0.78, 1.94**	VH GPR	**0.69, 30.37**	VH GPR	**0.85, 1.30**	VH GPR	**0.79, 20.92**
RFFE	**0.72, 2.29**	RFTB	**0.50, 37.93**	RFTB	**0.69, 1.91**	RFTB	**0.71, 26.23**
RFTB	**0.66, 2.51**	GBT	**0.48, 40.33**	GBT	**0.65, 2.16**	GBT	**0.66, 23.68**
GBT	**0.59, 2.80**	KRR	**0.33, 40.03**	RFFE	**0.63, 2.14**	RFFE	**0.55, 33.91**
KRR	**0.44, 3.09**	KSNR	**0.30, 43.61**	KRR	**0.48, 1.91**	KRR	**0.45, 34.38**
KSNR	**0.42, 3.10**	K-NNR	**0.21, 46.50**	KSNR	**0.41, 2.56**	K-NNR	**0.40, 35.71**
K-NNR	**0.30, 3.43**	RFFE	**0.25, 53.85**	K-NNR	**0.40, 2.57**	KSNR	**0.40, 35.84**

SOC data from 0–30 cm and 30–60 cm profiles are used

**Fig. 6:**
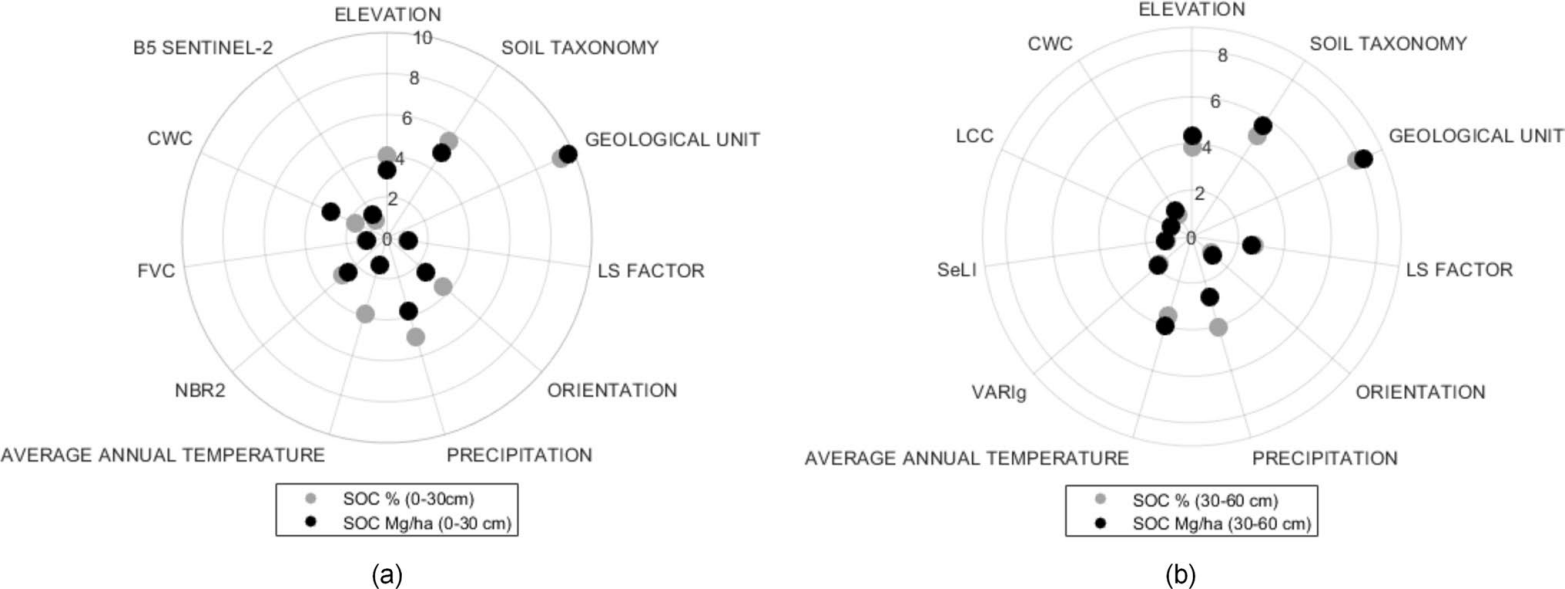
Variable relevance by sigma value σb of the GPR models. **a** SOC% model vs SOC Mg/ha model—0–30 cm profile, **b** SOC% model vs SOC Mg / ha model—profile 30–60 cm

**Fig. 7:**
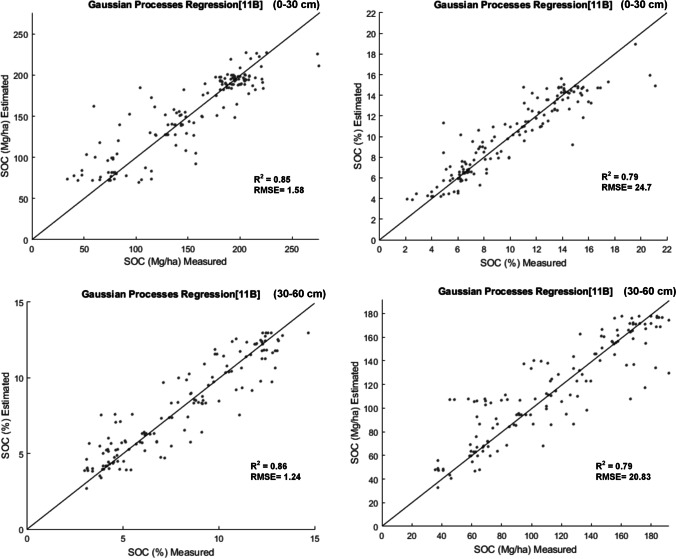
Scatterplot of the measured vs. estimated SOC values for the models: **a** SOC %- Profile 0–30 cm, **b** SOC Mg/ha – Profile 0–30 cm, **c** SOC % – Profile 30–60 cm, **d** SOC Mg / ha model—profile 30–60 cm. The 1:1 line is shown

Our results identified the most relevant variables for the prediction model in the 0**–**30 cm profile, being the variables Elevation, Soil Taxonomy, Geological Unit, LS Factor, Orientation, Precipitation, Average Annual Temperature, NBR2 Index, FVC, CWC, B5 the most significant in order to relevance. In the 0**–**60 cm profile they were: Elevation, Soil Taxonomy, Geological Unit, LS Factor, Orientation, Precipitation, Average Annual Temperature, VARIg Index, SeLI Index, LCC/C_ab_, CWC. Figure 6 shows the polar plots where the importance of the variables is shown in a comparative form through sigma value band (σb), based on their contribution within each SOC prediction model. From these results it is clear that Geological Unit, Soil Taxonomy, Precipitation and Elevation located near the edge of the polar plots were the most important variables for both profiles and in both units. Note that eight variables (i.e., Elevation, Soil Taxonomy, Geological Unit, LS Factor, Orientation, Precipitation, Average Annual Temperature, and CWC) define the SOC model in both soil profiles. Figure 7 shows the validation results of the models, with the distribution of the estimated SOC points vs. the measured ones being around the 1:1-line.

After calibrating the models with the most relevant variables, we obtained a high precision in SOC prediction, each model based on 11 variables or predictors. As a result, in the 0–30 cm profile, the SOC% prediction model has an R^2^ of 0.85, RMSE of 1.58% and the SOC model in Mg/ha reaches an R^2^ of 0.79, RMSE of 24.7 Mg/ha. The SOC model (in weight %) of 30–60 cm profile achieved an R^2^ of 0.86, RMSE of 1.24%, and the SOC model in Mg/ha reached an R^2^ of 0.79, RMSE of 20.83 Mg/ha.

**Fig. 8:**
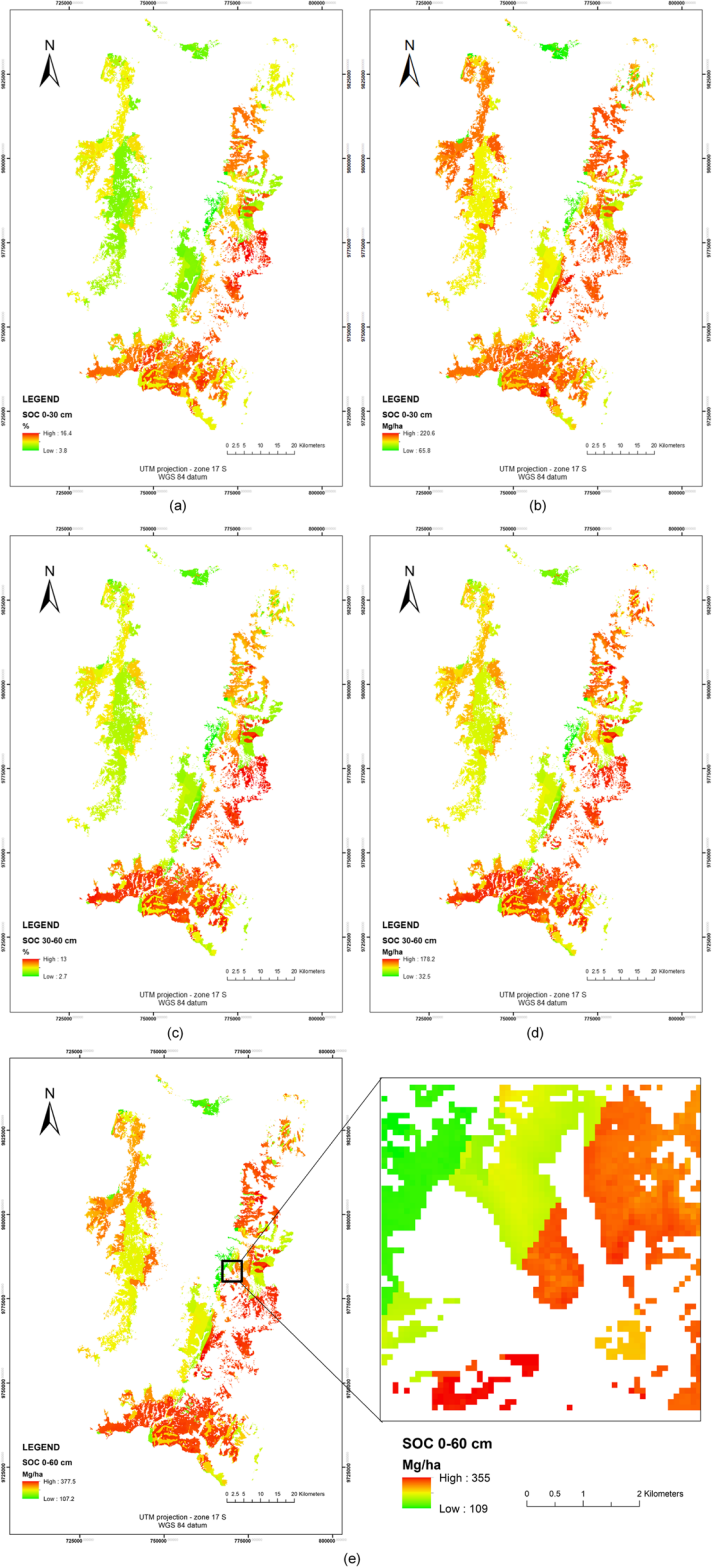
**a** Map of SOC prediction (in %)—0–30 cm profile, **b** Map of SOC prediction (in Mg/ha)—0–30 cm profile, **c** Map of SOC (in %)—30–60 cm profile, **d** Map of SOC (in Mg/ha)—30–60 cm profile, **e** Map of SOC (in Mg/ha)—0–60 cm profile. Zoom area is shown

Through the calibrated models, the prediction of the SOC values and the digital mapping were performed in both profiles, the results are shown in Fig. 8. It is found that, for the 0**–**30 cm profile, the values range between 3.8**–**16.4 SOC % and between 65.8**–**220.6 SOC Mg/ha. Regarding the 30–60 cm profile, the stored SOC is between 2.7**–**13 SOC % and between 32.5**–**178.2 SOC Mg/ha. The 30–60 cm profile shows lower SOC values compared to the upper profile, but, according to (Lefévre et al. 2017) and (Rumpel et al. 2012) the SOC in deep soil horizons would be more stable. SOC results in both profiles are added in order to obtain an accumulative SOC storage in the 0–60 cm Horizon, resulting in SOC values between 107.2 – 377.5 Mg/ha (see Fig. 8e). Also it is important to note that, in the zoom area (Fig. 8e) S2 predictors are able to detect local changes due to the spatial resolution of S2.

**Fig. 9:**
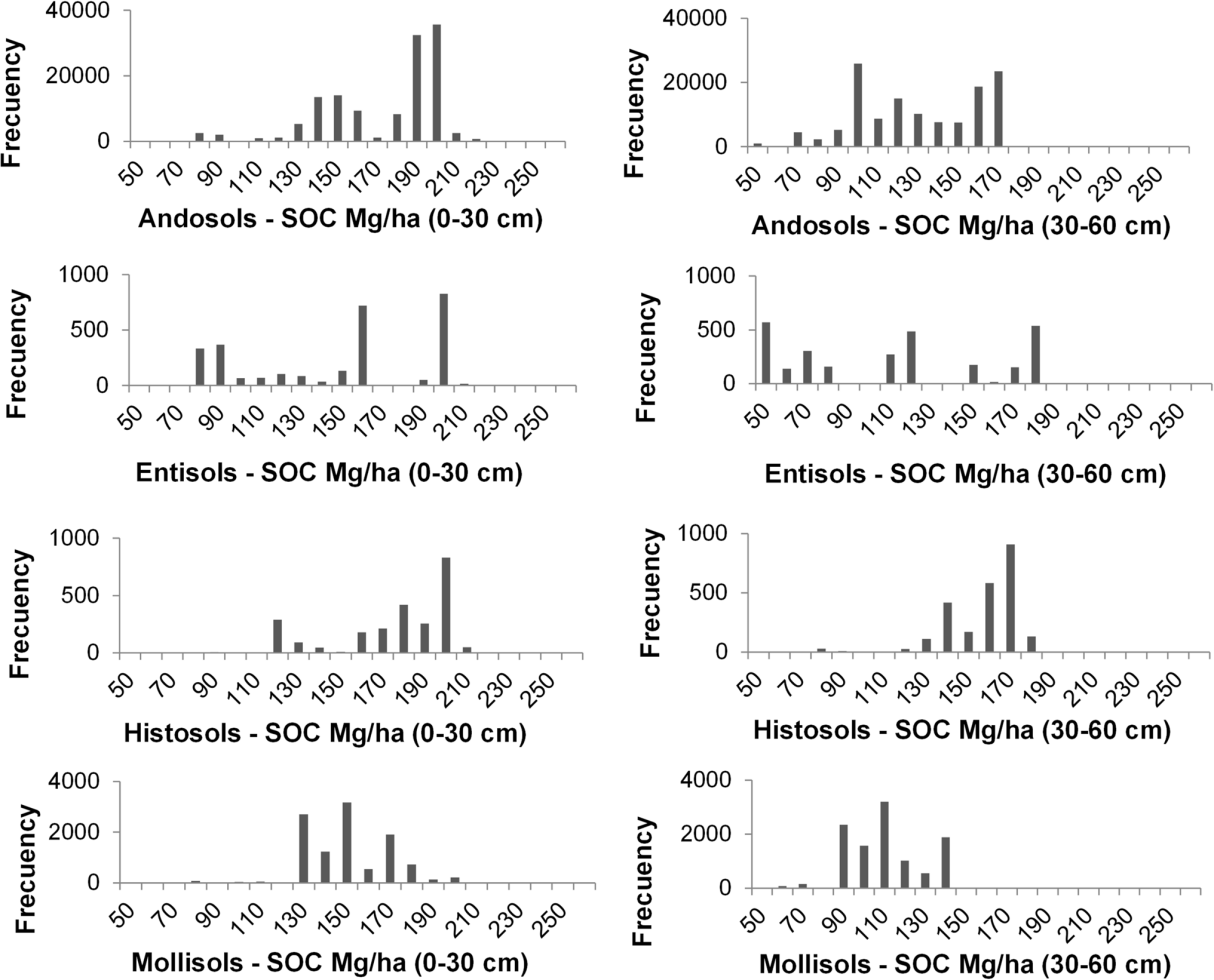
Histograms of SOC mapping distribution (0–30 cm profile and 30–60 cm profile) for each soil taxonomy, in Mg/ha for the whole study area

Due to the high importance of soil taxonomy variable the for SOC prediction, the results of SOC for all soil taxonomy type existing in the study zone (i.e., Andosols, Entisols, Histosols and Mollisols) were obtained and compared; we can detect variations over SOC distribution on the different soil types (see Fig. 9) (additional data are given in Online Resource 1). Andosols predominate in the study area with a share of 89%. Characterized by presenting a melanic epipedon, they are dark soils where SOM forms a stable complex with materials non-crystalline inorganic substances. Hence the decomposition of SOM is delayed, allowing the accumulation of SOC (Buytaert et al. 2005), which is observed in the results with high carbon concentrations between 190 and 200 Mg C/ha (see Fig. 9). Histosols represent a lower percentage of the study area (1.7%), and may have lower or higher carbon concentrations depending on the folic and histic epipedon values (Hribljan et al. 2016). Mollisols are soils with slightly acidic pH originating a mollic epipedon (Moreno et al. 2016), these conditions increase the mineralization of organic matter, predominantly values ​​around 150 Mg C/ha. They cover approximately a 7.4% of the study area and are located in areas that have a higher temperature regime and an ustic moisture regime, their parent material is influenced by the Paute geological formation, which has the presence of basaltic rocks that originate soils. In the case of Entisols present in 1.9% of the study area, these soils show the lowest SOC concentrations of the results; located on higher slopes, they are young and in some cases they are subjected to erosion processes. Figure 9 shows high SOC values within the group of Entisols, which may be related to inclusions of Andosols since these landscapes have a complex spatial distribution. The results of SOC storage predicted seem to be in concordance with the soil type but it is important to note the existence of some SOC values outside the usual SOC values, especially in Entisols. We speculate that this might be due to problems of delimitation of the types of soils of the existing national cartography, where in the soil taxonomy map, the soils in analysis share limits, likewise in the cartography pedological units are allowed with up to 15% inclusions of other soils (SIGTIERRAS 2012). Figure 9 shows the complexity of the retrieval, even so, our SOC models are able to find almost gaussian-like distributions of the parameter for some soil types due to the appropriately sampling strategy.

## Discussion

Earlier studies of SOC estimation for different study areas using S2 and other spaceborne platforms report a R^2^ below 0.7 (Nocita et al. 2013; Mirzaee et al. 2016; Steinberg et al. 2016; Vaudour et al. 2019). The comparative performance results of the regression methods analysed (see Table 4)—using the same variables to predict SOC, and using the same SOC database—show that GPR obtained better results compared to the other models, and this improvement corresponds only to the model. A previous study using Random Forest Regression for the same study area using Landsat 8 (OLI and TIRS sensors) obtained 0-30 cm profile obtained a R2 of 0.82 and RMSE of 1.72 (for SOC%) and a R2 of 0.77 and RMSE of 25.8 (for SOC in Mg/ha) (Ayala Izurieta et al. 2021); comparing RFTB with GPR in this study we obtain that GPR accuracy is better that RFTB in a 15% for SOC% and 26% for SOC Mg/ha in the 0–30 cm profile, and for the 30–60 cm profile, GPR model is better that RFTB in a 17% and 9% corresponding to SOC% and SOC in Mg/ha respectively. This study obtained similar results of SOC predicted in 0–30 profile, but with different spectral variables using S2 images, therefore we obtained an increase of the spatial resolution and also new SOC indicators for the model of SOC prediction were found (i.e., FVC, CWC, LCC/Cab and the SeLI index). It shows the importance of spectral indices into the models. The results indicate that topography has a higher influence on SOC at finer spatial scales (Adhikari et al. 2020), which is indicated by the high importance obtained with the elevation, orientation and LS Factor as SOC predictors. Soil formation conditions on páramo depend on three main factors which are climate, bedrock and the age of the soils (Mena et al. 2000). In general, the páramo soils are vast SOC reservoirs where the capacity of SOC storage into the soils depend on soil types and their morphologic, physical and chemical parameters. These parameters are approached with Geological Unit, Soil Taxonomy, Precipitation and Average Annual Temperature predictors (see Fig. 6), also the SOC mapping results revealed that the SOC distribution is strongly guided by Geological Unit and Soil Taxonomy predictors (see Fig. 8).

Our results are according to (Minaya et al. 2016), due to the higher SOC stocks are found in the low and mid catchments, which could be associated to higher content of soil silt fraction. Our SOC predicted values have variations comparing profile 0-30 cm and profile 30-60 cm. Thompson et al. (2021) found changes over bulk density occur with increase of soil depth, and also SOC changing in function of land use. The land use change is related with a change of soil properties to SOC stock and stabilization on Andean ecosystems of Ecuador (Tonneijck et al. 2010). Therefore, a possible linking of SOC stocks and the change in soil texture would be interesting to hold study, in order to detect the change patron to different soil profiles.

S2 variables also contributed to the improved prediction, such as the biophysical variables FVC, CWC and LCC/C_ab_, from reflectivity of S2-band 5 (705 nm) and SeLI index, considering them as new SOC predictors. These variables used at a resolution of 10 m, give more resolution to mapping results for zones with sudden alterations in SOC storage produced by mainly anthropic actions or sudden environmental changes, having obtained a fine scale on SOC mapping results. FVC variable results from normalized difference vegetation index (NDVI) values of highly dense vegetation (NDVI_v_) and bare soil (NDVI_s_) (Montandon and Small 2008; Jiapaer et al. 2011), this variable provides more information to the model than NDVI alone, which had no impact on the SOC prediction model. FVC can describe the quality of herbaceous páramo vegetation and its possible changes, also considering that anthropic intervention over the ecosystem affects the endemic vegetation. The páramo soils and its vegetation have high capacity to retain water (Podwojewski and Poulenard 2004), this seems to be proven with the CWC (Verrelst et al. 2020) as the biophysical variable that shows the highest importance to the SOC prediction models, giving more sensitivity mainly to the types of vegetation based on the soils conditions since, taking account the perennial vegetation (MAE 2013) owns of páramo ecosystem. LCC or C_ab_ give important information for the results in the profile 30–60 cm, and these results are in line with studies that found potential for this variable to model the carbon cycle and the chemical process into the plants (Osborne and Raven 1986; Carter 1994; Qian and Liu 2020). An advantage of the MSI from Sentinel 2 is the location of Band 5 (705 nm) corresponding to the Near Infrared (NIR) edge (ESA 2015). Here, the reflectance produced by the vegetation varies by an effect to the leaf chlorophyll and leaf structure, the change is more subtle in the case of dry vegetation or soil, allowing a good discrimination for changes in land cover and vegetation cover. In the same way, the SeLI index exploits the red-edge region for low-saturating absorption sensitivity to photosynthetic vegetation (Pasqualotto et al. 2019) providing predictive power to the SOC model for the 30–60 cm profile and for the accumulative 0–60 cm profile.

## Conclusions

SOC storage in both soil profiles (0–30 cm and 30–60) were estimated using GPR models with an accuracy of 85% for the 0–30 cm profile and 86% for the 30–60 cm profile, based on commonly used and novel SOC predictors. With the inclusion of five new S2-derived SOC predictors, i.e., FVC, CWC, LCC/C_ab_, S2 band 5 (705 nm) and the SeLI index, we were able to improve the estimation results and increase its spatial resolution. Products with high spatial resolution like S2, favor the SOC mapping resolution. Apart from classic SOC predictors (Geological Unit, Soil Taxonomy, Precipitation and Average Annual Temperature), CWC emerged as the most relevant biophysical variable. Therefore, SOC reserves are correlated with the aboveground biophysical characteristics and changes over it.

The retrieval works adequately, the results evidenced at high detail the high SOC storage in the vast and poorly accessible study area. The methodology presented here could be used and applied to obtain the digital SOC mapping over other areas of herbaceous páramo ecosystem and also over ecosystems with similar conditions and characteristics.

Between all MLRA, GPR stood out to predict SOC in complex areas, even outperforming methods such as RFR, KRR, NN, etc. A high precision was achieved, with the identification of the important characteristics to the SOC prediction model. Therefore, it was possible to find 14 SOC predictors linked to SOC sequestration in order of importance for their contribution to the optimization of the resulting model. In the 0–30 cm profile the order of relevance was: Elevation > Soil Taxonomy > Geological Unit > LS Factor > Orientation > Precipitation > Average Annual Temperature > NBR2 Index > FVC > CWC > B5. In the 0–60 cm profile: Elevation > Soil Taxonomy > Geological Unit > LS Factor > Orientation > Precipitation > Average Annual Temperature > VARIg Index > SeLI Index > LCC/C_ab_ > CWC.

## Supplementary Information

Below is the link to the electronic supplementary material.

Supplementary file1 (XLSX 8511 kb)

## Data Availability

The datasets generated during and/or analysed during the current study are available as supplementary material.

## Abbreviations

SOC: Soil organic carbon
SOM: Soil organic matter
OM: Organic matter
S2: Sentinel-2
MSI: Multi-spectral instrument
ESA: European Space Agency
EUMETSAT: European Organization for the Exploitation of Meteorological Satellites
FAPAR: Fraction of Absorbed Photosynthetically Active Radiation
LAI: Leaf Area Index
FVC: Fractional Vegetation Cover
LCC/C_ab_: Leaf Chlorophyll Content
CWC: Canopy Water Content
MLRA: Machine Learning Regression Algorithms
RFFE: Random Forest Fitensemble
RFTB: Random Forest TreeBagger
GBT: Gradient Boosting/Boosted Trees
KNNR: K-nearest neighbors regression
NN: Neural Network
KRR: Kernel ridge Regression
KSNR: Kernel signal to noise ratio
VH-GPR: Kernel-based method Variational Heteroscedastic Gaussian Processes Regression
GP: Gaussian Process
GPR: Gaussian Processes Regression
GloVis: Global Visualization Viewer
DEM: Digital Elevation Model
TOA: Top Of Atmosphere
BOA: Bottom Of Atmosphere
NDMI: Normalized Difference Moisture Index
SAVI: Soil-Adjusted Vegetation Index
WDRVI: Wide Dynamic Range Vegetation Index
EVI2: Enhanced Vegetation Index 2
NDWI: Normalized Difference Water Index
VARIg: Visible Atmospherically Resistant Vegetation Index green
NDSI: Normalized Difference Snow Index
BI: Bare Soil Index
NDMI: Normalized Difference Moisture Index
NBR: Normalized Burn Ratio
NBR2: Normalized Burn Ratio 2
SeLI: LAIgreen Index
ARTMO: Automated Radiative Transfer Model Operator
